# Shoulder instability in the middle-aged and elderly patients: Pathology and surgical implications

**DOI:** 10.4103/0973-6042.79791

**Published:** 2010

**Authors:** Joe de Beer, Deepak N. Bhatia

**Affiliations:** Cape Shoulder Institute, Platekloof, Cape Town, South Africa; 1 Department of Orthopaedic Surgery, Seth GS Medical College and King Edward VII Memorial Hospital, Parel, Mumbai, India

Shoulder instability is frequently encountered in young patients and commonly follows sporting injuries. There are well-recognized aspects pertaining to labral tears and bone loss leading to increased risks for re-dislocations and high recurrence rates in the young. However, shoulder instability in the middle-aged and elderly patients is an under-recognized problem, with a different pathological spectrum and specific surgical implications.[1,2] This article outlines the principles that guide management of this entity.

When dealing with shoulder instability, the term “middle-aged” may be applicable to patients who are in the age group 40-55 years, and “elderly” patients are those above 60 years of age. In these groups, less force is required to dislocate the shoulder due to the fact that the ligaments are less elastic and rupture easily. Labral lesions are less common, and capsular tears occur more frequently. Associated tuberosity and humeral neck fractures occur more frequently. Greater tuberosity fractures associated with anterior dislocations in the elderly occur due to a shear and/or compression mechanism and not due to avulsions. Therefore, bone loss on the humeral head is often considerable, and simple reduction of the greater tuberosity may not result in the insertion of the rotator cuff at a normal height. Surgical reduction of these fractures, therefore, should include a bone graft for the humeral head defect, to restore the height of the tuberosity and the lever arm of the rotator cuff. Neurovascular injuries are more common, especially of the axillary nerve, leading to deltoid paresis. Other brachial plexus injuries may also occur.

Missed dislocations are encountered more frequently in the middle-aged and elderly patients. Osteoporosis of humeral or glenoid bone occurs in missed anterior or posterior dislocations, and significant wear occurs rapidly; massive loss of bone can become a major reconstructive challenge, should surgery be indicated. In missed chronic anterior dislocations, pseudo-aneurysms may occur, and surgical reduction may be accompanied by a vascular catastrophe, with high morbidity and mortality. Presence of a vascular surgeon during such procedures is advisable. Posterior dislocations are missed more frequently; these are often regarded as frozen shoulders by the medical personnel due to lack of motion, especially limited external rotation.

Dislocations in elderly patients are often accompanied by tears of the rotator cuff. Reduction of a dislocated shoulder should be accompanied with a rapid decrease in pain. If the pain persists after 2 weeks, a rotator cuff tear should be strongly suspected. Early repair of these tendons is indicated to avoid long-term functional disability. Biceps lesions frequently accompany rotator cuff tears in these patients; subluxations, dislocations or ruptures of the biceps tendon should be treated with a biceps tenodesis or tenotomy. A painful and stiff shoulder may occur following a dislocation and closed reduction in the elderly. A possible mechanism is a capsular tear, which heals through a process of capsulitis. The prognosis of this is usually excellent, with a self-limiting tendency. The possibility of pre-existing cartilage wear of the joint (or accelerated wear following the injury) should be considered, and this would imply a poor outcome after reduction due to the osteoarthritic process.

In conclusion, shoulder instability in the middle-aged and elderly patients presents with features different from those of the more common type seen in the young and active patients. Detailed clinical and radiological evaluations are necessary to diagnose the structural lesions. Surgical repair of the associated pathological lesions is necessary to prevent recurrence and future functional deficits.
